# Acknowledgment to the Reviewers of *Clocks* & *Sleep* in 2022

**DOI:** 10.3390/clockssleep5010005

**Published:** 2023-01-28

**Authors:** Clocks & Sleep Editorial Office

**Affiliations:** MDPI AG, St. Alban-Anlage 66, 4052 Basel, Switzerland

High-quality academic publishing is built on rigorous peer review. *Clocks* & *Sleep* was able to uphold its high standards for published papers due to the outstanding efforts of our reviewers. Thanks to the efforts of our reviewers in 2022, the median time to first decision was 19 days and the median time to publication was 55 days. Regardless of whether the articles they examined were ultimately published, the editors would like to express their appreciation and thank the following reviewers for the time and dedication that they have shown *Clocks* & *Sleep*:
Anderson, KirstieMainieri, GretaArai, ToshiroManiaci, AntoninoAsaoka, ShoichiMassart, AlainAten, SydneyMatsushita, DaisukeBaptista, PeterMederos Crespo, SaraBarry, Elaine S.Michalek-Zrabkowska, MonikaBhatt, VrushankMizuno, KohBikov, AndrasMorales Suárez-Varela, María M.Borisenkov, Mikhail F.Mota-Rolim, Sergio A.Bramoweth, Adam D.Mukherjee, DidhitiBrzecka, AnnaMulin, EmmanuelBurek, KatarzynaMuranaka, TomoakiButlewski, MarcinNavarro, RaulBüttner-Teleagă, AntjeNevin, Remington L.Castelli, LuciaO’Keeffe, KarynChen, ChenghaoOchi, GentaCiavarella, DomenicoOginska, HalszkaCosta, RaquelOlson, Jay A.De Berardis, DomenicoOtelea, Marina RuxandraDe Zeeuw, JanPageat, PatrickDevine, JaimePandey, Atul KumarDouma, LaurenPati, AtanuElder, Greg J.Pawlas, KrystynaGhiani, CristinaPecor, Keith W.Gordijn, MarijkePedrazzoli, MarioGujski, MariuszPerrier, JoyHaba-Rubio, JosePesonen, Anu-KatriinaHarangi, MariannPilon, MathieuHarbison, Susan T.Plaza, GuillermoHarfmann, BriannaPutilov, ArcadyHariston, IlanaReiter, Andrew M.Hartanto, AndreeRipperger, Jürgen A.Hearn, TimothySantamaria, JoanJaramillo-Morales, Osmar AntonioSantos, Francisco Eroni Paz DosJung, AlesiaSchmidt, ChristinaKantas, DimitriosSchredl, MichaelKim, HyeyunSharman, RachelKim, JinkwanSingh, ShailendraKochetova, OlgaSkeldon, Anne C.Kohyama, JunSong, DanKolomeichuk, SergeySpiegelhalder, KaiKompotis, KonstantinosStefani, OliverKoritala, Bala S.C.Szewczyk-Golec, KarolinaKrishnan, JishnuWięckiewicz, MieszkoLi, XiangWu, XiaoyanLi, Yao-ChuenWu, YeLok, RenskeYamazaki, YudaiLopes, FernandYoung, MichaelLópez-Gil, José FranciscoZeitzer, JamieLowden, ArneZhang, ChaoLucas, RobertZobaer, M.S.


